# Pigment Epithelium‐Derived Factor (PEDF)‐Based Therapy Induced Photoreceptor Survival by Stabilizing Choroidal Neovessels in a VEGF Overexpression CNV Rat Model

**DOI:** 10.1096/fj.202501704RR

**Published:** 2025-10-09

**Authors:** Alexander V. Tschulakow, Lei Xi, Ulrich Schraermeyer, Sylvie Julien

**Affiliations:** ^1^ Centre for Ophthalmology, University Medical Center Eberhard Karls University of Tuebingen Tübingen Germany; ^2^ Institute for Ophthalmic Research, University Medical Center Eberhard Karls University of Tuebingen Tübingen Germany; ^3^ OcuTox GmbH, Preclinical Drug Assessment Hechingen Germany

**Keywords:** AMD, anti‐VEGF therapy, CNV, CNV rat model, PEDF, VEGF

## Abstract

Pathological choroidal neovascularization (CNV), characterized by abnormal leaky vessel formation, can lead to vision impairment or blindness. Current standard treatment, anti‐vascular endothelial growth factor (VEGF) therapy, while effective, can be associated with severe local and systemic side effects and resistance in some patients, necessitating novel therapeutic approaches. Evidence from wet age‐related macular degeneration (AMD) donor eyes and patients suggests that functional, quiescent CNV can aid photoreceptor survival. Therefore, stabilizing functional neovessels rather than removing them could offer an effective alternative for treating pathological CNV. Pigment epithelium‐derived factor (PEDF), a multifunctional protein with antiangiogenic, neuroprotective, and vessel‐stabilizing properties, is a promising candidate for this approach. The efficacy of intravitreally injected PEDF protein, alone or in combination with bevacizumab, was investigated and compared to bevacizumab monotherapy in a VEGF overexpression‐induced CNV rat model using in vivo imaging and histology. Following PEDF + bevacizumab treatment, a significant reduction in CNV thickness was observed, comparable to bevacizumab monotherapy. Notably, only PEDF and PEDF + bevacizumab treatments significantly preserved retinal thickness and enhanced photoreceptor survival at the CNV site. Ultrastructural analysis revealed that PEDF and PEDF + bevacizumab treatments increased CNV vessel lumina, stabilized these vessels by reducing the thickness of the extracellular matrix and refining its structure, and enhanced vessel fenestration and pericyte coverage, making PEDF‐based therapy more effective than bevacizumab monotherapy. These findings suggest that supporting the maintenance of functional CNV vessels using PEDF may represent a novel and innovative strategy for the treatment of wet AMD.

## Graphical abstract

Stabilizing choroidal neovessels offers a novel treatment‐option for pathological CNV. Pigment epithelium‐derived factor (PEDF), a multifunctional protein with vessel‐stabilizing properties, is a promising candidate for this approach. Its efficacy with and without bevacizumab was tested in a VEGF overexpression CNV rat model. PEDF, alone or in combination with bevacizumab preserved retinal thickness and enhanced photoreceptor‐survival at the CNV‐site, stabilized the choroidal neovessels (CNV), improved their extracellular matrix structure and enhanced fenestration and pericyte coverage.

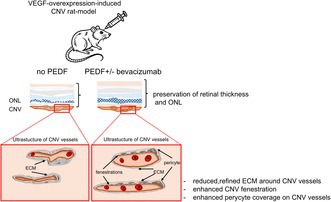

## 1 Introduction

Choroidal neovascularization (CNV) is characterized by the growth of newly formed blood vessels from the choriocapillaris through Bruch's membrane into the subretinal space. While this may be regarded as a healing mechanism aimed at replacing lost choriocapillaris and overcoming insufficient blood supply, pathological changes of these vessels can lead to stasis, edema, and even retinal detachment [1]. Pathological CNV is a typical symptom of several ocular diseases such as degenerative myopia, central serous chorioretinopathy, diabetic retinopathy, and wet age‐related macular degeneration (AMD) [2, 3, 4].

In a previous study, analyzing the ultrastructure of CNV vessels in AMD donor eyes, we found pathological changes like reduced or even collapsed lumina, thickened poorly organized extracellular matrix, vessels with dying or dead endothelia, known as “ghost capillaries” and vessels with microvilli‐like projections of the endothelium forming a labyrinth‐like structure reaching into the vessel's lumen—features associated with impaired perfusion and tissue degeneration [1, 5].

Despite its clinical significance, the exact mechanisms underlying healthy CNV formation and its pathological progression remain unclear.

Conventional CNV treatments—laser coagulation, surgery, radiation, photodynamic therapy, and intravitreal injections of anti‐vascular endothelial growth factor (VEGF) drugs—aim to suppress neovessel growth. Currently, anti‐VEGF agents are the standard therapy for CNV treatment, showing benefits including visual acuity improvement and CNV regression in acute wet AMD [6, 7, 8], as well as in other CNV disorders such as myopic CNV and diabetic retinopathy [9, 10]. However, some patients are initial nonresponders to anti‐VEGF therapy, while others develop acquired resistance over time [11, 12]. Moreover, there is a growing body of evidence linking long‐term anti‐VEGF therapy to the development or exacerbation of geographic atrophy (GA), a symptom of advanced AMD [13, 14, 15, 16, 17, 18], and potential systemic side effects, such as stroke and myocardial infarction [19]. Therefore, a CNV therapy solely based on anti‐VEGF agents is not optimal, necessitating the development of more refined therapeutic approaches.

A recent study using OCT‐angiography revealed a prevalence of non‐leaky, quiescent CNV in fellow eyes of patients with unilateral exudative CNV. In these eyes, a higher perfusion rate was identified, indicating that functional CNV vessels maintain blood supply to the overlying tissues [20]. These results support earlier findings from our group in donor eyes with late‐stage wet AMD, demonstrating that healthy functional CNV vessels support the survival of the overlying retinal pigment epithelium (RPE) and photoreceptors [5, 21] and later observations in AMD patients [22].

Therefore, our innovative approach aims to stabilize functional CNV vessels rather than completely suppress CNV formation. A promising candidate for such a new CNV treatment is pigment epithelium‐derived factor (PEDF). As a natural antagonist of VEGF, PEDF balances VEGF‐driven neovascularization [23]. Moreover, it has been shown to stabilize the structure and function of normal blood vessels [24]. In 2006, a phase I clinical trial using an adenoviral human PEDF vector for neovascular AMD was performed. Unfortunately, in the trial, PEDF was regarded only as an antiangiogenic agent, and therefore only changes in the CNV lesion size were analyzed. PEDF showed only modest antiangiogenic activity; additionally, the immunogenicity of the adenoviral vectors was observed [25]. Thus, this approach was not further pursued.

More recent approaches using less immunogenic adeno‐associated virus (AAV) vectors allowed the expression of PEDF and anti‐VEGF‐A microRNAs [26] or PEDF and the complement system regulator sCD59 [27]. Both multigenic AAV vector approaches have shown a significant reduction of CNV in laser‐induced CNV mouse models and are promising for the treatment of neovascular AMD; however, further development is required before these strategies can be translated into clinical practice [28].

In a mouse wound healing model, PEDF exhibited antifibrotic, wound‐healing, and vessel‐stabilizing effects [29]. Therefore, therapeutic administration of PEDF should also mitigate pathological CNV formation and have beneficial effects on the quality of neovessel growth and structure [30]. In an ex vivo model of hypoxia using rat eyes, we demonstrated that treatment with PEDF protein significantly increased the vessel quality by stabilizing the structure of choriocapillaris vessels, keeping their lumina open, and inhibiting labyrinth‐like capillary formation. Moreover, it significantly induced the survival of neuroretinal cells [31].

Similar benefits were observed in an oxygen‐induced retinopathy model, where PEDF protein alone or combined with VEGF antagonists significantly increased vessel quality by reducing pathological artery tortuosity and pathological neovascularization [32].

In the present study, we evaluated PEDF therapy in a human VEGF overexpression‐induced CNV rat model previously established in our lab [33]. A special emphasis was laid on the analysis of CNV vessel quality related to photoreceptor survival using electron microscopy. The effect of the treatments on CNV progression/regression was investigated using in vivo imaging and histology.

## 2 Materials and Methods

### 2.1 Ethics Statement

This study utilized experiments conducted on rats. All experiments were performed with the approval of the Animal Experimentation Committee of the University of Tuebingen “Regierungspraesidium Tuebingen” (AK 01/21G). All animals were handled in accordance with the German Animal Welfare Act and were under the control of the animal protection agency and the supervision of veterinarians at the Eberhard‐Karls University of Tuebingen. The study did not involve any human experiments.

### 2.2 Animals

Six‐week‐old female Long Evans (LE) rats were purchased from Janvier Labs (Le Genest‐Saint‐Isle, France). Female rats were selected due to their docile behavior. In total, 15 rats were used in this study.

### 2.3 Proof of Efficacy Study in a VEGF Overexpression CNV Rat Model

#### 2.3.1 Experimental Design

**FIGURE 1:**
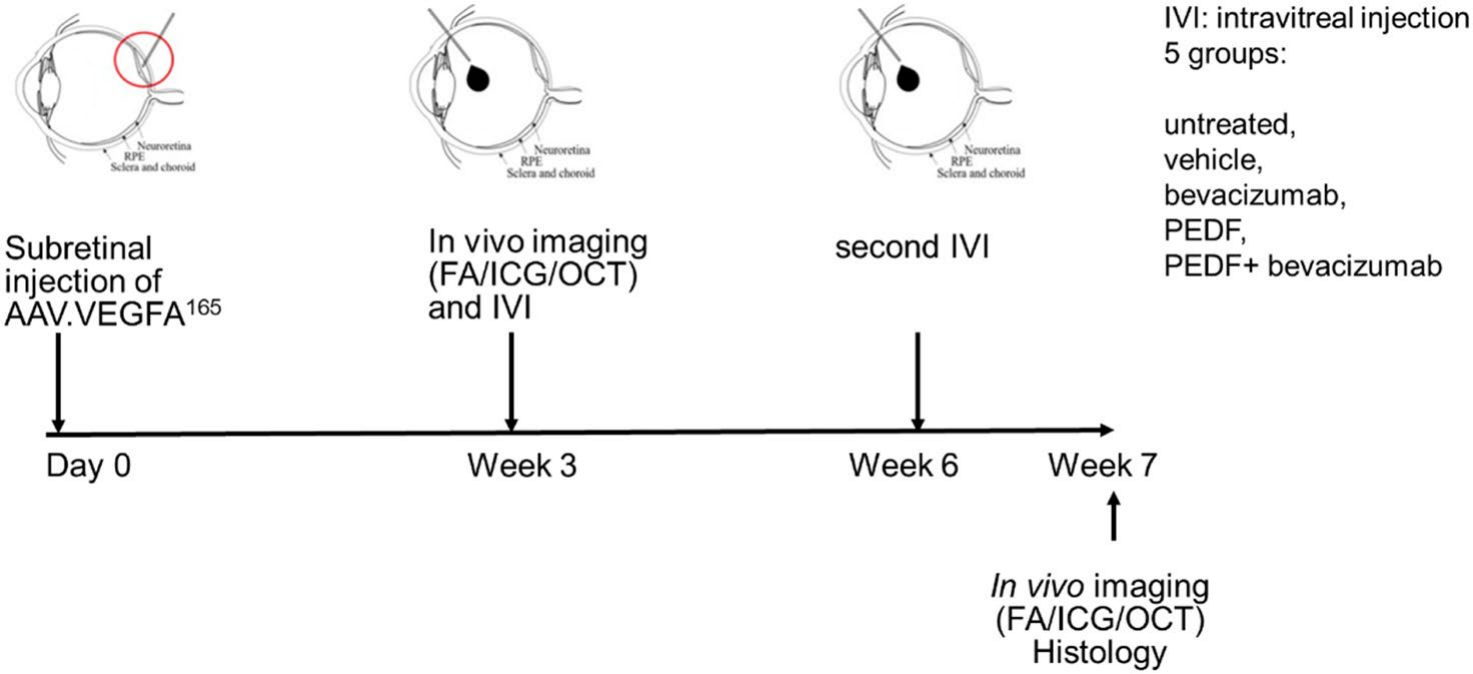
Study design of the proof of efficacy study in a VEGF overexpression CNV rat model. CNVs were induced by subretinal injection of AAV.VEGF^165^ in both rat eyes. Pre‐treatment FA/ICG/OCT examinations were performed three weeks after vector injection. Intravitreal injections (IVT) were administered at 3 and 6 week post‐vector injection. The treatment groups: Untreated, vehicle, bevacizumab, PEDF and PEDF + bevacizumab are indicated in the upper right corner (*n* = 6 eyes/group). At 7 weeks post‐vector injection, after the final FA/ICG/OCT analysis, the rats were sacrificed, and their eyes fixed for histology.

The experimental design is shown in Figure 1. CNV was induced by subretinal injections of AAV.VEGF^165^ in both eyes of the 15 rats (30 eyes) as described in [33].

In vivo examinations were performed at the third week after subretinal injection of the vector in order to investigate the transduction's success with VEGF vector, that is, the formation of CNV. Intravitreal injections with bevacizumab, human PEDF protein, a combination of PEDF protein with bevacizumab (PEDF+bevacizumab), and vehicle were administered immediately after the first in vivo examination and a second time 3 weeks later (3 weeks and 6 weeks post vector injection, respectively) as depicted in Table 1. Bevacizumab was purchased at the Pharmacy of the University of Tuebingen, Germany. The human PEDF protein was kindly provided by Prof. Dr. S. Kochanek (Department of Gene Therapy, University of Ulm, Germany). As we already demonstrated the efficacy of 10 μg of PEDF protein per eye in an ex vivo ischemia model [31] and a similar dose in an OIR retinopathy model [32], we decided to use the dose of 10 μg/eye in this study.

**TABLE 1:** Overview of the treatment modalities, number of injected animals/eyes, injected volume and amount of the treatment substances, for each of the injection time points (3 and 6 weeks after AAV.VEGF vector injection) are indicated.

Treatment	Intravitreal injection 3 and 6 weeks after VEGF‐AAV vector injection	
	Number of animals/eyes	Injected volume, amount
Untreated	3/6	—
Vehicle PBS	3/6	4 μl
Bevacizumab	3/6	4 μl, 50 μg
Human PEDF protein	3/6	4 μl, 10 μg
Human PEDF protein/bevacizumab	3/6	4 μl, 10 μg/50 μg

The rats were sacrificed after the final in vivo analysis at the seventh week post‐VEGF vector injection. All eyes were enucleated and fixed for light and electron microscopy analysis.

#### 2.3.2 Subretinal Injection of AAV.VEGF‐A^165^ Vector for CNV‐Induction

To induce CNV formation, the same vector and procedure previously described by us in [33] was used. Briefly, in the rats, narcosis was induced using an intraperitoneal injection of a mixture of three components (0.005 mg fentanyl, 2 mg midazolam, and 0.15 mg of medetomidine/kg body weight). The pupils were dilated with 1 to 2 drops of Medriaticum (Pharmacy of the University of Tuebingen, Germany), and a drop of the topical anesthetic Novesine (OmniVision, Puchheim, Germany) was applied. Methocel (OmniVision, Puchheim, Germany) eye drops were used to prevent drying of the eyes.

First, the sclera was opened with a 25 G needle close to the limbus, then 2 μL of vector suspension (2 μL PBS containing 2 × 10^9^ virus particles AAV‐VEGF, max. possible dose) was injected subretinally using a 10 μL NanoFil syringe with a NanoFil 34 G blunt needle (World Precision Instruments). Topical antibiotic eye drops Gentamicin‐POS (Ursapharm, Saarbrücken, Germany) were applied after the injection. The narcosis was neutralized by subcutaneous injection of the antidote (0.12 mg naloxon, 0.2 mg flumazenil, 0.75 mg atipamezol/kg body weight).

#### 2.3.3 Intravitreal Injections

Narcosis, pre‐ and post‐care procedures were performed identically to those for the subretinal injections. For intravitreal injection, a small incision was made into the conjunctiva at the outer corner of the eyes. A volume of 4 μL of the corresponding substances (Table 1) was injected intravitreally through the hole using a 10 μL NanoFil syringe with a NanoFil 34 G beveled needle (World Precision Instruments). After the injection, the needle remained in the eye for an additional 3 to 4 s to reduce reflux before removal.

#### 2.3.4 In Vivo Imaging (SLO, FA, ICG and OCT)

Scanning laser ophthalmoscopy (SLO), fluorescein angiography (FA), indocyanine green angiography (ICG), and optical coherence tomography (OCT) were performed three (pre‐treatment to assess CNV formation) and seven (final examination) weeks post‐VEGF vector injection using a Spectralis HRA + OCT device (Heidelberg Engineering, Heidelberg, Germany). To adapt it for analyses in rats, a +78D double aspheric lens (Volk Optical Inc., Mentor, OH 44060, USA) was placed directly on the outlet of the device.

In vivo analyses were performed under the same narcosis as the subretinal and intravitreal injections. The pupils were fully dilated using 1 to 2 drops of Medriaticum (Pharmacy of the University of Tuebingen, Germany). An additional custom‐made +7 diopter (dpt) contact lens was placed directly on the rat's eyes.

The ICG dye (250 μL (VERDYE, 5 mg/mL, Diagnostic Green)) was injected into the tail vein, and the fluorescein dye (Alcon 10% (1/10 dilution), 250 μL) was injected subcutaneously. SLO/OCT was performed approximately 2 to 5 min after injection for early phase and approximately 15 to 20 min later for late phase angiography imaging. Note that angiography in rats takes longer than in patients, as the SLO/OCT machine is calibrated for human eyes, requiring careful positioning of the rat's eyes.

#### 2.3.5 Measurement of the Maximal Thickness of the CNV and the Overlying Retinal Thickness Using OCT

**FIGURE 2:**
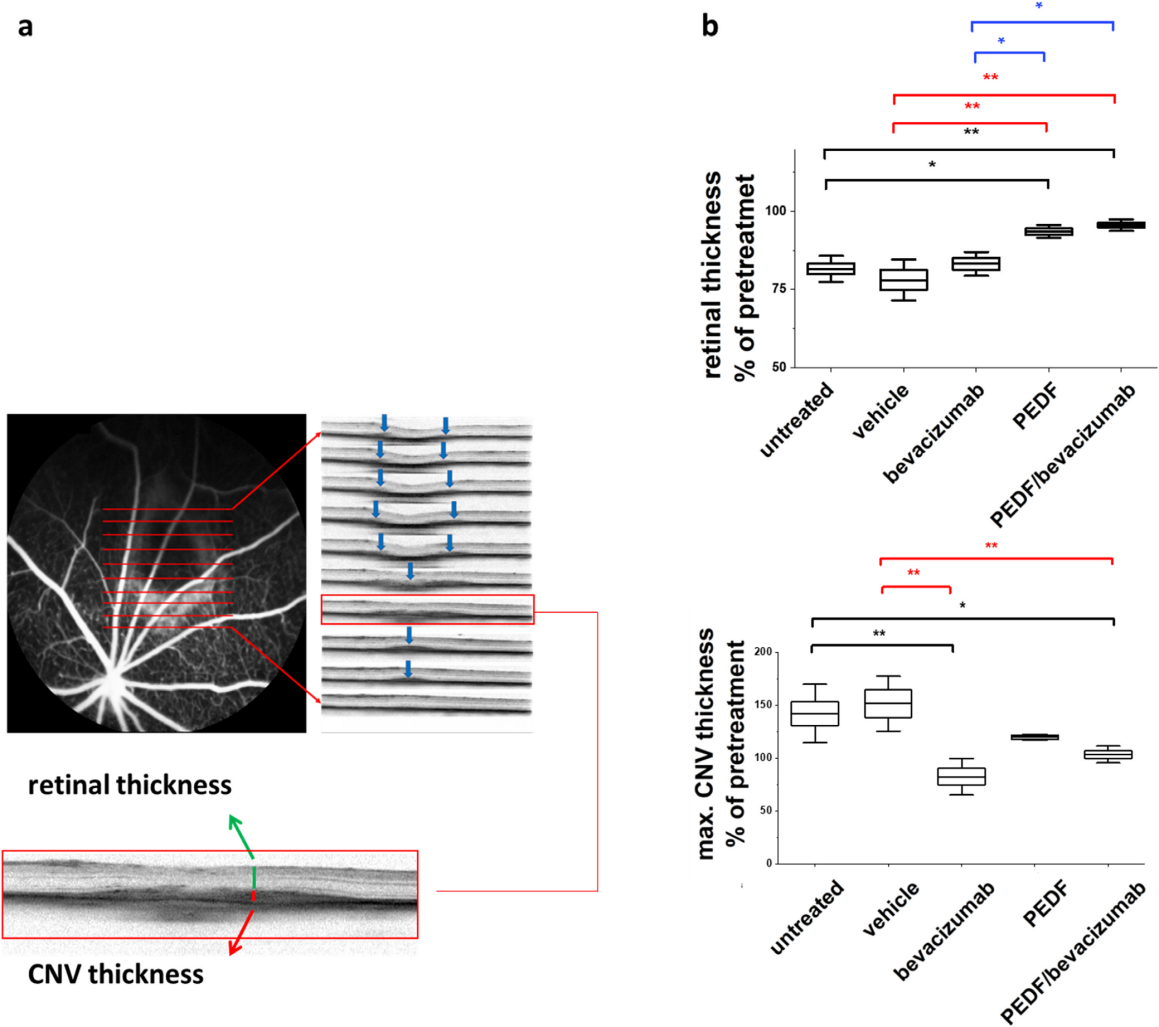
OCT measurement of the maximal thickness of the CNV and the retinal thickness over that area. (a) Principle of the OCT analysis. OCT scans were performed across the CNV ring‐shaped area (red lines). On the OCT scan (red frame), the maximal CNV thickness (red) and the retinal thickness over the CNV area (green) were manually measured. (b) Upper panel: Measurement of the retinal thickness over the maximal thickness of the CNV area 7 weeks after VEGF vector injection, expressed as a percentage, normalized to pre‐treatment values (3 weeks after VEGF vector injection), *n* = at least 3 eyes per group. Lower panel: Measurement of the maximal CNV thickness 7 weeks after VEGF vector injection, expressed as a percentage, normalized to pre‐treatment values (3 weeks after VEGF vector injection). *n* = at least 3 eyes per group. Results compared to the untreated control are shown in black, to the PBS control in red and to bevacizumab in blue, *p < 0.05, **p < 0.001. ANOVA with Sidak‐Holm post‐test.

The same method as reported previously in [33] was used. The principle of the measurement is demonstrated in Figure 2a. During OCT analysis, the back of the eye was screened by the operator and the area of the CNV was determined; images were captured in the form of a volume scan. In cases of cataract or strong eye movements, no measurements could be performed. On these OCT scans, the maximal thickness of the CNV and the retinal thickness over that area were manually measured using ImageJ software. The values from the measurement performed 3 weeks after vector injection (pre‐treatment) for each eye served as internal standards (100%). The values from subsequent measurements at week seven for each individual eye were normalized against these pre‐treatment values and calculated as percentages.

#### 2.3.6 Quantification of the of the ONL Area Over the CNV Area on Histological Slides

**FIGURE 3:**
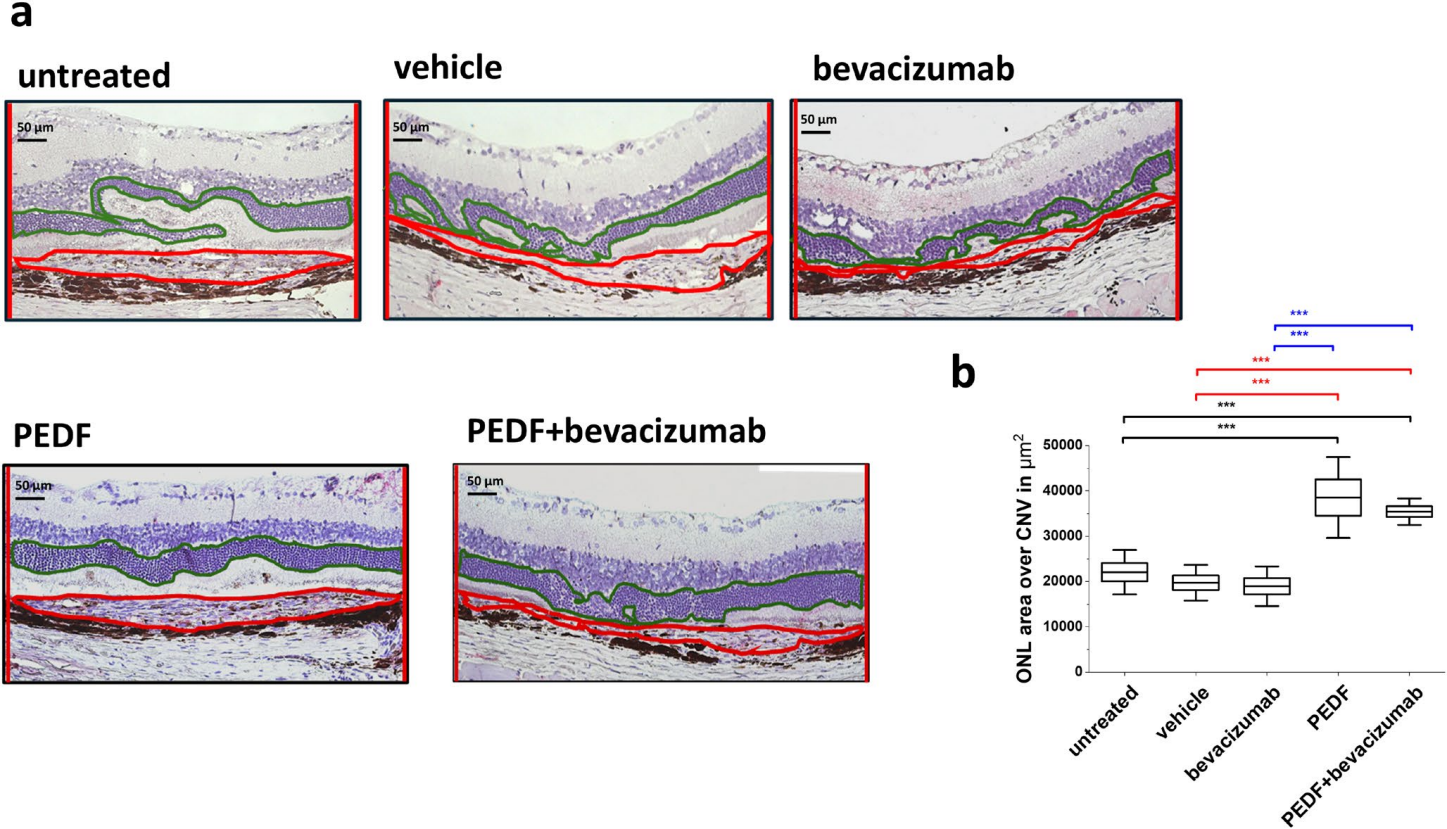
Quantification of the ONL area over the CNV area on histological slides. (a) Principle of the analysis. LM image showing the selected CNV‐area (red) and ONL area over the CNV (green) in the thickest part of the CNV. (b) Analysis of the area of the outer nuclear layer (ONL) over the CNV‐area 7 weeks after VEGF vector injection, *n* = 3 eyes per group. Results compared to the untreated control are shown in black, to the PBS control in red and to bevacizumab in blue, ***p < 0.0001, ANOVA with Sidak‐Holm post‐test.

Directly after the final in vivo analysis, the rats were sacrificed, enucleated, and half of the eyes were fixed in 4.5% formalin (Roti Histofix, Carl Roth, Karlsruhe, Germany), followed by embedding in paraffin using standard protocols. They were then cut into 4 μm thick sections. The CNV‐containing sections were stained with eosin/hematoxylin and examined using a light microscope. Figure 3 shows representative histological images for each treatment. The area of the outer nuclear layer (ONL) over the CNV was measured in the thickest part of the CNV using ImageJ software.

#### 2.3.7 Electron Microscopy

Contralateral eyes were fixed in 5% glutaraldehyde in 0.1 M cacodylate buffer (pH 7.4) overnight at 4°C. The CNV areas were identified, using the SLO‐angiography images for orientation, and then cut. These samples were post‐fixed with 0.1% osmium tetroxide, stained with saturated uranyl acetate, dehydrated, and embedded in Epon using standard protocols. Ultrathin sections (0.05 μm) were stained with lead citrate. Examinations were performed using a Zeiss 900 transmission electron microscope (Zeiss, Jena, Germany).

#### 2.3.8 Ultrastructural Examination of the CNV Areas

**FIGURE 4:**
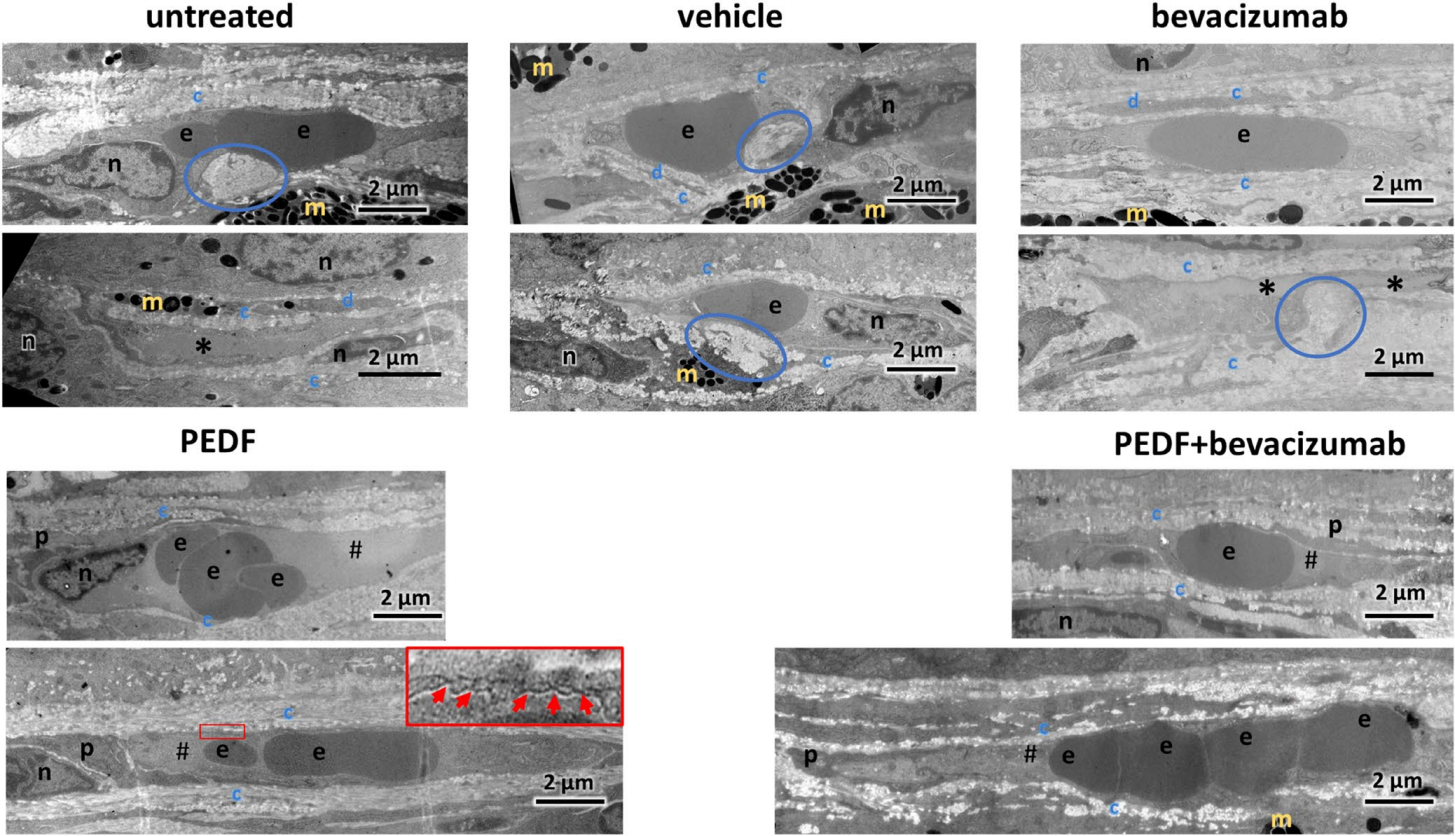
Representative electron micrographs (7000× magnification) of CNV vessels from each treatment group. CNV vessels in the untreated, vehicle‐treated, and bevacizumab‐treated eyes exhibited similar ultrastructural characteristics: Reduced lumina, with many vessels appearing collapsed (*), surrounded by thick, clustered (circled in blue) ECM layers (blue c, collagen layer; blue d, layer of electron dense material). These vessels mostly lacked pericytes and fenestrations. In contrast, PEDF‐ and PEDF+bevacizumab treated eyes showed CNV vessels with larger and open lumen (#). The surrounding ECM layer was thinner and less clustered (ECM cluster, circled in blue; blue c, collagen layer; blue d, layer of electron dense material). Additionally, most vessels in these eyes were associated with pericytes (p) and exhibited fenestrations (red arrows). e, erythrocytes; n, nucleus; t, thrombocytes; yellow m, melanin granules.

Electron micrographs covering the entire CNV area at a magnification of 3000× were generated for two eyes of each treatment group and used to create overview images of these areas, so‐called multi‐image alignments (MIA) (Supplement 2). These MIAs were screened for CNV vessels, and electron micrographs at a magnification of 7000× of all detected CNV vessels were taken (Figure 4). Additionally, for comparison, images of choriocapillaris vessels outside the CNV area from two untreated animals were taken at the same magnification (7000×). These images were closely examined, and features for detailed quantitative analyses were determined.

#### 2.3.9 Measurement of the Cross‐Section Area of CNV Vessel Lumina

**FIGURE 5:**
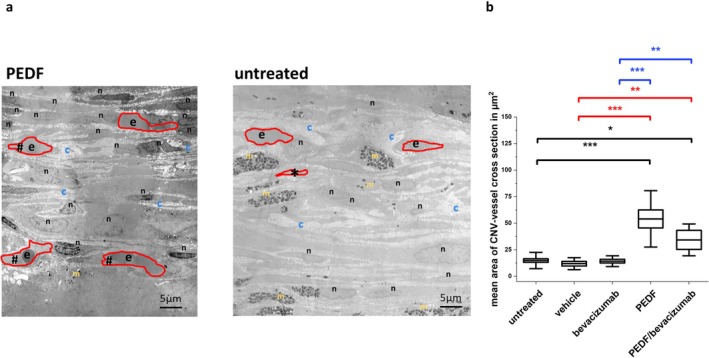
Analysis of the cross‐section areas of the CNV vessels. (a) Left: Electron micrograph (3000×) showing a part of the CNV area from a PEDF‐treated eye. Right: Electron micrograph showing a part of the CNV area from an untreated eye. The cross‐section areas of the vessel lumina are highlighted in red. #, open lumen; *, collapsed lumen; blue c, collagen layer; n, nucleus; e, erythrocytes; yellow m, melanin granules. (b) Measurement of CNV vessel lumina cross‐sections. Shown are mean ± SD, *n* = 2 eyes per group. Results compared to the untreated control are shown in black, to the PBS control in red, and to bevacizumab in blue, *p < 0.05, **p < 0.001, ANOVA with Sidak‐Holm post‐test.

The cross‐sectional areas of the CNV vessels' lumina were manually measured on MIAs (3000×) using the ImageJ software as indicated in Figure 5a.

#### 2.3.10 Measurement of Extracellular Matrix (ECM) Thickness Around Vessels

**FIGURE 6:**
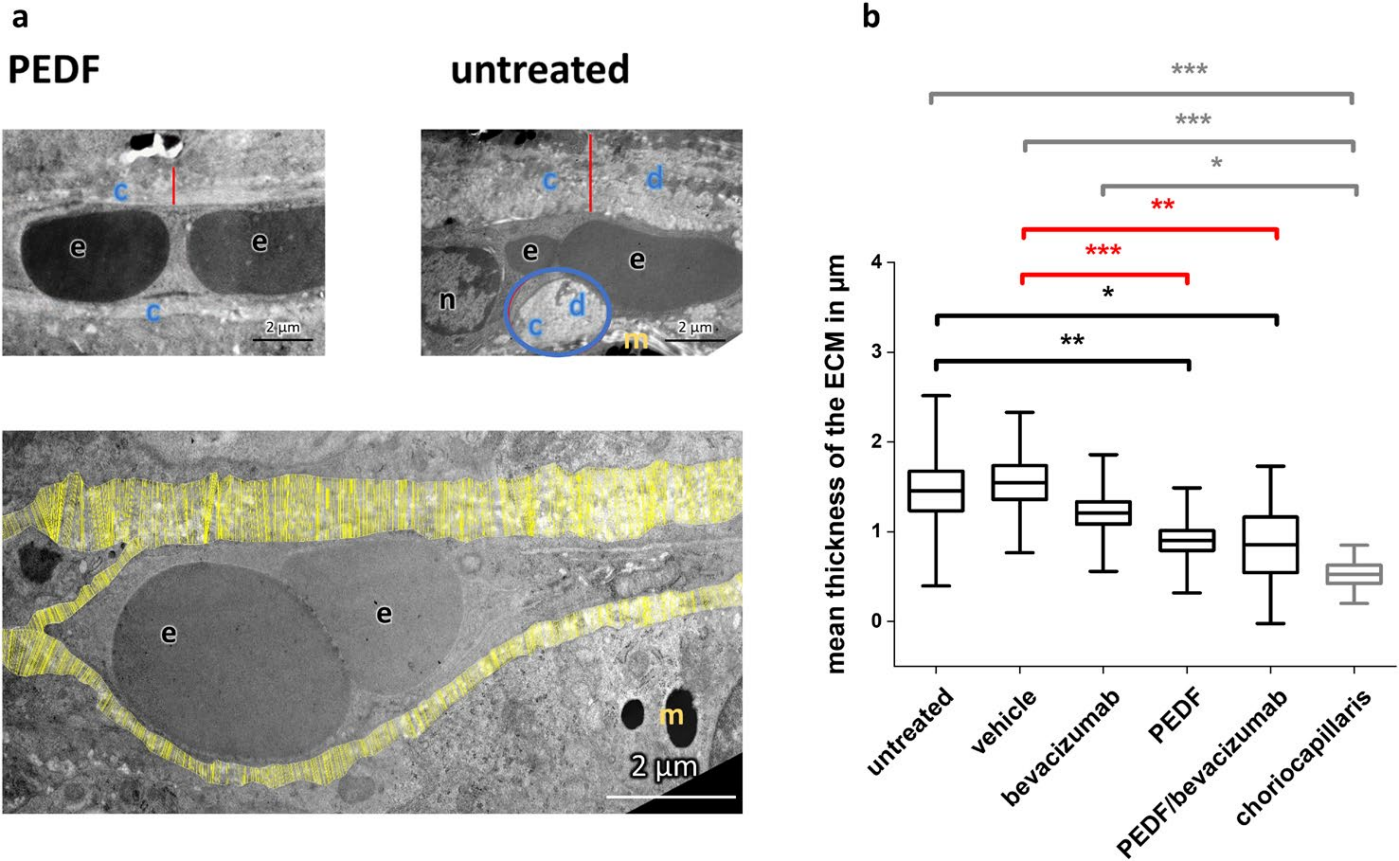
Measurement of ECM thickness around the CNV vessels. (a) Upper panel: Electron micrographs (7000×) of a CNV vessel from a PEDF‐treated eye (left) and an untreated eye (right). Red lines indicate the area of maximal ECM thickness. A large ECM‐cluster is circled in blue; blue c, collagen layer; blue d, layer of electron dense material; n, nucleus; e, erythrocytes; yellow m, melanin granules. Lower panel: Principle of the analysis. ECM mean thickness around the CNV vessels was measured using ImageJ (InteredgeDistance_v1.4_ImageJMacro). Each yellow line represents a single measurement. (b) Measurement of ECM thickness around CNV vessels. Shown are mean ± SD, *n* = 2 eyes per group. Results compared to the untreated control are shown in black, to the PBS control in red, and to the choriocapillaris group in gray, *p < 0.05, **p < 0.001, ***p < 0.0001, ANOVA with Sidak‐Holm post‐test.

On electron micrographs, the ECM around the vessels can be clearly determined as a distinct, coherent area composed of electron‐lucent (white) layers (collagen) with embedded electron‐dense (darker) layers. For the analysis of the ECM thickness around the CNV vessels using electron micrographs (7000×), a semi‐automatic ImageJ macro‐based approach was used (InteredgeDistance_v1.4_ImageJMacro). The principle is shown in Figure 6a (lower part). Additionally, the ECM thickness around choriocapillaris vessels outside the CNV area from two untreated animals was measured.

#### 2.3.11 Quantification of Vessels With Pericytes and Vessels With Fenestrations on EM‐Images

**FIGURE 7:**
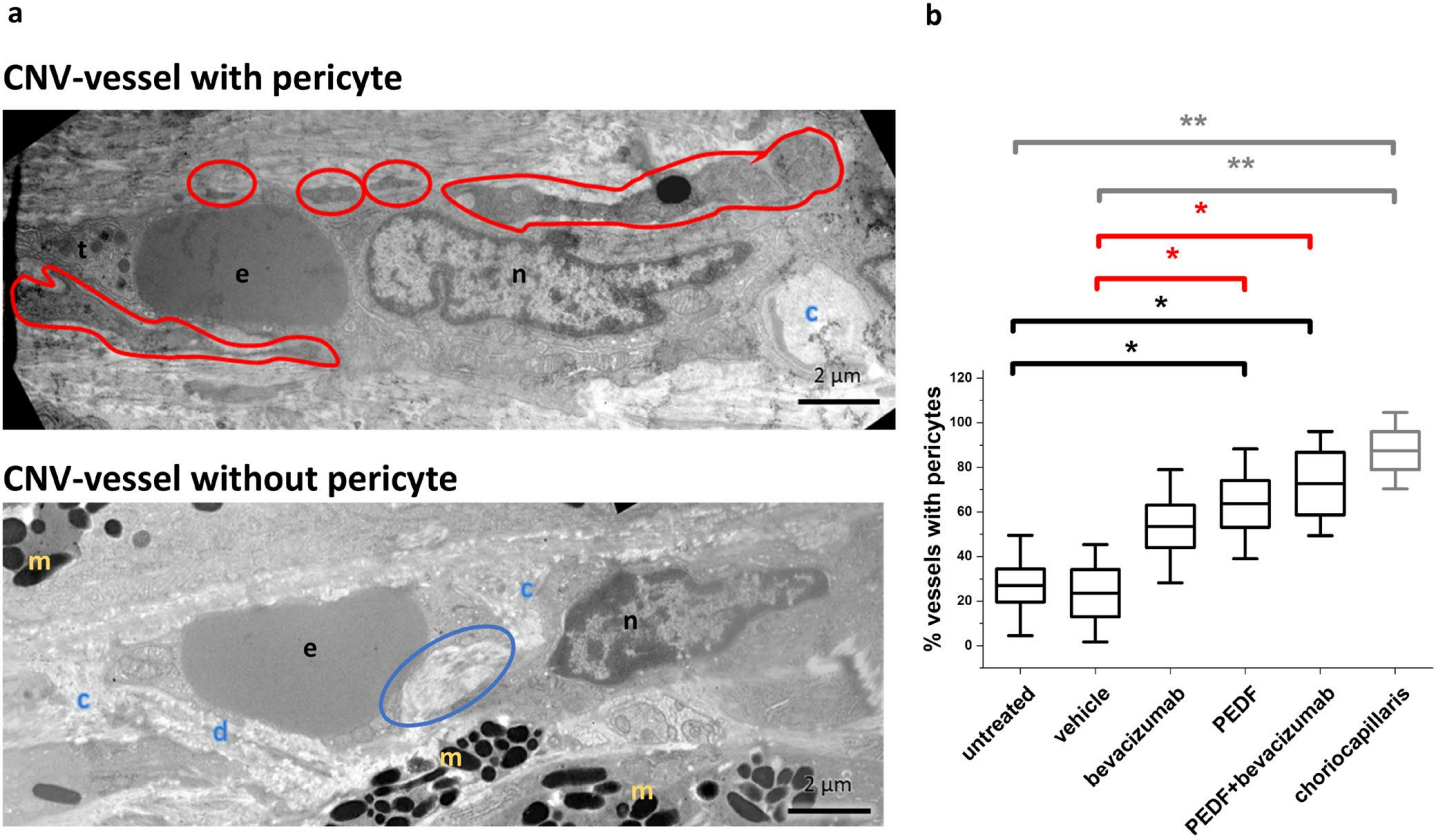
Quantification of vessels with pericytes. (a) Electron micrograph of a CNV vessel associated (upper part) and not associated (lower part) with a pericyte (pericyte framed in red, note several cross‐section areas from pericyte filopodia), a large ECM‐cluster is circled in blue; blue c, collagen layer; blue d, layer of electron dense material; n, nucleus; e, erythrocytes; t, thrombocyte; yellow m, melanin granules. (b) Quantification of CNV vessels and choriocapillaris vessels associated with pericytes. Shown are mean ± SD, *n* = 2 eyes per group. Results compared to the untreated control are shown in black, to the PBS control in red, and to the choriocapillaris group in gray, *p < 0.05, **p < 0.001, ANOVA with Sidak‐Holm post‐test.

**FIGURE 8:**
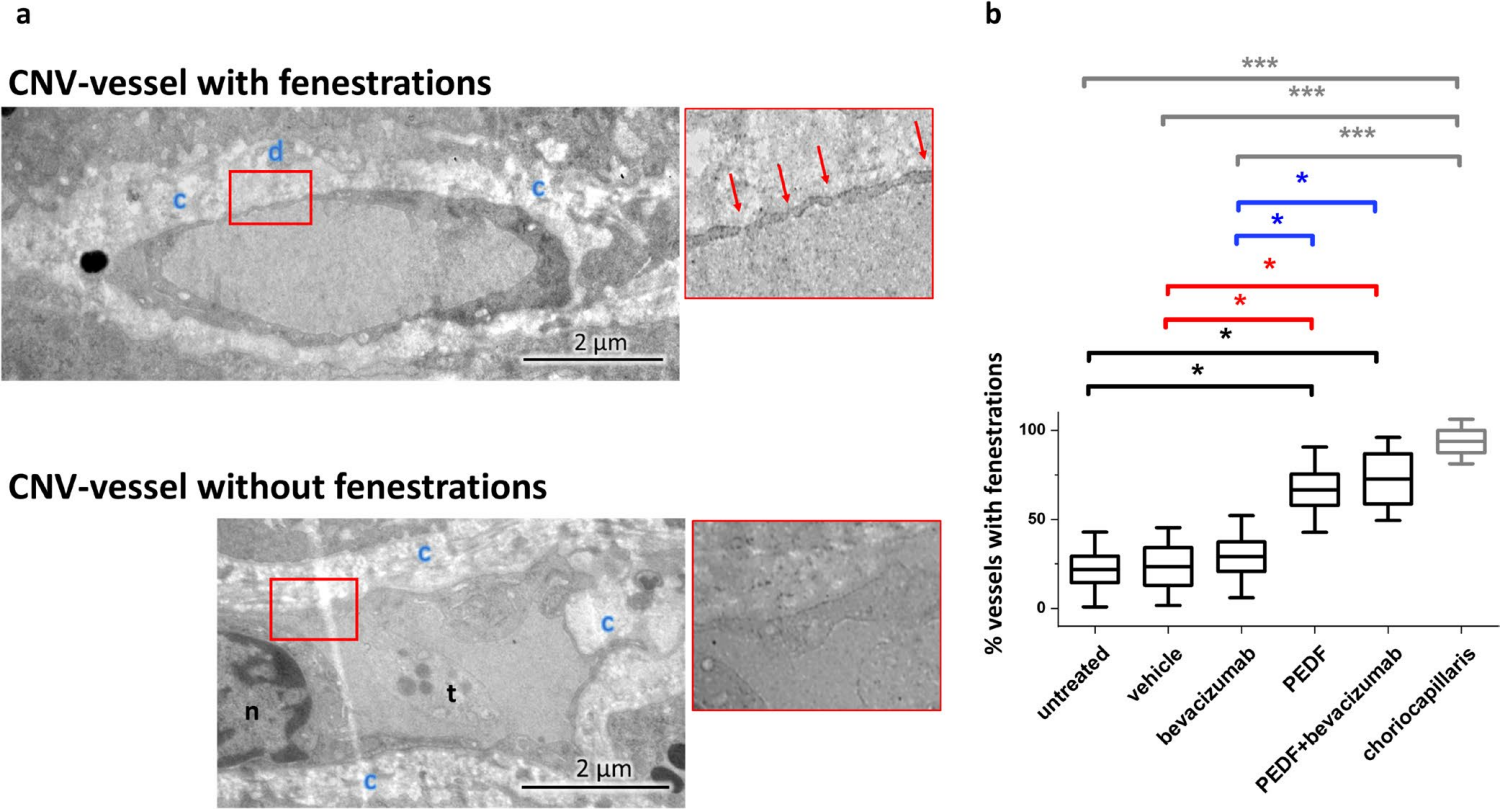
Quantification of fenestrated CNV vessels and choriocapillaris vessels. (a) Electron micrographs of a CNV vessel with (upper part) and without fenestrations (lower part). Close‐up‐images of fenestrated and non‐fenestrated areas (red frame) are shown next to the figures. Fenestrations are indicated with red arrows; blue c, collagen layer; blue d, layer of electron dense material; n, nucleus; t, thrombocyte; e, erythrocytes; yellow m, melanin granules. (b) Quantification of fenestrated CNV vessels and choriocapillaris vessels. Shown are mean ± SD, *n* = 2 eyes per group. Results compared to the untreated control are shown in black, to the PBS control in red, to bevacizumab in blue and, to the choriocapillaris group in gray, *p < 0.05, ***p < 0.0001, ANOVA with Sidak‐Holm post‐test.

The CNV vessel images (7000×) were screened for the presence of pericytes. Pericytes were identified by their localization close to the capillary wall, within the endothelial ECM and using typical morphological characteristics [34, 35] such as prominent nuclei, sparse cytoplasm, and poorly developed cell organelles. In some cases, cross‐sections of filopodia could be observed as well (Figure 7a). The CNV vessel images were then examined for the presence of fenestrations. Examples of fenestrated endothelium are shown in Figures 4 and 8a (framed in red).

#### 2.3.12 Statistics

Statistical analyses were performed using ORIGIN PRO software (Version 2024 10.1). The results were presented as means ± standard deviation (SD). For statistical analyses of multiple groups, ANOVA for multiple comparisons with a Sidak‐Holm post‐test was used. Differences were considered statistically significant at a *p*‐level < 0.05.

## 3 Results

### 3.1 Proof of Efficacy Study in a VEGF Overexpression CNV Rat Model

#### 3.1.1 In Vivo Imaging of CNV‐Development

Three weeks after AAV.VEGF vector injection, all 30 eyes displayed a characteristic pattern in both FA and ICG angiographies, with a central hypofluorescent (dark) area surrounded by a hyperfluorescent (bright) ring (Figure 2a, Supplement 1), showing a 100% transduction efficacy of the VEGF vector. This ring‐shaped form of the CNV area was caused by the subretinal vector injection which induced a reversible retinal detachment [33]. The FA and ICG signals correlated well (Supplement 1). The CNV‐area appeared in both angiographies as an ill‐defined hyperfluorescent area without leakage or pooling of dyes in the late phase, corresponding to quiescent CNV [36]. The scanning laser ophthalmoscopy (SLO) results correlated well with optical coherence tomography (OCT) analyses, which revealed pronounced subretinal CNV‐lesions indicated by hyper‐reflective (dark) areas (Figure 2a).

#### 3.1.2 Measurement of the Maximal Thickness of the CNV and the Overlying Retinal Thickness Using OCT

Seven weeks post‐VEGF vector injection (i.e., 1week after the second intravitreal treatment) the maximal CNV thickness was significantly reduced in the bevacizumab (90.45% ± 10.6% of pretreatment)‐ and PEDF+bevacizumab (104.3% ± 11.9% of pretreatment)‐treated eyes compared to the vehicle (152.2% ± 24.2% of pretreatment)‐treated and untreated (123.5% ± 31.6% of week 3) eyes (ANOVA, *p < 0.05, **p < 0.001, ***p < 0.0001) (Figure 2b). At the same time point, a significant retinal thickness rescue was measured in the PEDF (93.5% ± 2.05% of pretreatment)‐ and PEDF+bevacizumab‐treated (95.2% ± 1.85% of pretreatment) eyes, compared to the bevacizumab (83.2% ± 3.8% of pretreatment)‐, vehicle (78.05% ± 6.5% of pretreatment)‐treated and untreated (81.6% ± 4.1% of week 3) eyes (ANOVA, *p < 0.05, **p < 0.001) (Figure 2b).

#### 3.1.3 Quantification of the ONL Area Over the CNV Area on Histological Slides

Seven weeks after the vector injection, the area of the ONL over the CNV area in the PEDF (38 538 ± 8929 μm^2^)‐ and PEDF+bevacizumab (35 415 ± 2905 μm^2^)‐treated eyes was significantly higher than in the bevacizumab (18 971 ± 4379 μm^2^)‐, vehicle (19 725 ± 3926 μm^2^)‐treated, and untreated (22 065 ± 4912 μm^2^) eyes (ANOVA, p*** < 0.0001), indicating more surviving photoreceptor cells after PEDF and PEDF+bevacizumab treatments in the CNV area (Figure 3b).

#### 3.1.4 Ultrastructural Examination of the CNV Areas

The CNV areas were investigated by electron microscopy (EM). Representative images for all treatment‐ and control groups are shown in Figure 4. Significant differences were observed in the structure of the CNV vessels and surrounding extracellular matrix (ECM), as well as in the presence of pericytes and fenestrations. Based on these observations, quantitative analyses of these features were performed.

#### 3.1.5 Measurement of the Cross‐Section Area of CNV Vessel Lumina

The mean CNV vessel lumina in the untreated (14.86 ± 7.67 μm^2^), vehicle (11.9 ± 5.63 μm^2^)‐ and bevacizumab (14.22 ± 5.09 μm^2^)‐treated eyes was significantly reduced compared to the CNV vessel lumina of the PEDF (48.01 ± 18.83 μm^2^)‐ and PEDF+bevacizumab (40.28 ± 15.01 μm^2^)‐treated eyes (ANOVA, *p < 0.05, **p < 0.001, ***p < 0.0001) (Figure 5b).

#### 3.1.6 Measurement of the ECM Thickness Around Vessels

The ECM around the CNV vessels was significantly thicker in the untreated (1.46 ± 0.7 μm) and vehicle (1.54 ± 0.52 μm)‐treated eyes compared to that around the choriocapillaris (0.53 ± 0.21 μm) vessels (ANOVA, ***p < 0.0001), and the CNV vessels of the PEDF (0.9 ± 0.38 μm)‐ and PEDF+bevacizumab (0.86 ± 0.58 μm)‐treated eyes (ANOVA, *p < 0.05, **p < 0.001). The ECM around the CNV vessels in the bevacizumab (1.2 ± 0.43 μm)‐treated eyes was significantly thicker compared to that around the choriocapillaris vessels (ANOVA, *p < 0.05).

#### 3.1.7 Quantification of Vessels Associated With Pericytes

Significantly fewer CNV vessels were associated with pericytes in untreated (27.03% ± 22.5%) and vehicle‐treated (23.53% ± 21.9%) eyes compared to the choriocapillaris vessels (87.5% ± 17.07%) and CNV vessels from PEDF (63.6% ± 24.6%) and PEDF+bevacizumab (72.7% ± 23.35%)‐treated eyes (ANOVA, *p < 0.05, **p < 0.001) (Figure 7b).

#### 3.1.8 Quantification of Vessels With Fenestrations

Quantification of vessels with fenestrations showed that in the untreated (21.8% ± 21%), vehicle (24% ± 21.85%)‐ and bevacizumab (36.64% ± 24.4%)‐treated eyes, significantly fewer fenestrated CNV vessels were present, compared to the choriocapillaris vessels (93.8% ± 12.5%) and CNV vessels of the PEDF (66.7% ± 23.9%)‐ and PEDF+bevacizumab (72.7% ± 23.35%)‐treated eyes (ANOVA, *p < 0.05, ***p < 0.0001) (Figure 8b).

## 4 Discussion

The aim of this study was to evaluate the efficacy of PEDF protein alone or in combination with bevacizumab for CNV treatment compared to the current standard treatment, anti‐VEGF therapy.

Since PEDF protein's half‐life is similar to that of currently used anti‐VEGF agents, we performed intravitreal injections twice, at 3 and 6 weeks after CNV induction, to mirror the monthly anti‐VEGF injections used in patients. We used our previously established rat model for a treatment‐naive quiescent CNV without leakage [33]. However, our model mimicked CNV vessels with reduced lumen and collapsed vessels (Figures 4 and 5a), features typically observed in patients with CNV [1, 5]. Therefore, an analysis of a potential stabilizing effect of PEDF on leaky vessels could not be performed here and will be the aim of future studies using an appropriate model. In the present model, the eyes exhibited ongoing VEGF expression and CNV formation with a significant increase in lesion thickness over at least 9 weeks after vector injection. For the present experiment, this indicates that the different treatments competed with ongoing VEGF expression. Indeed, in the untreated and vehicle‐treated eyes, the CNV thickness steadily increased while the thickness of the overlying retina decreased over time (Figure 2b). Using only OCT, the higher retinal thickness measured in the PEDF‐ and the PEDF/bevacizumab groups can't be clearly associated with cell survival and might be caused by other reasons like, for example, swelling of the retina. But the analysis of the histological slides (Figure 3b), which showed a thicker ONL area over the CNV in the PEDF‐ and PEDF/bevacizumab groups, indicated photoreceptor survival.

This effect can be partly attributed to the known neuroprotective activity of PEDF [37], particularly its protective effects on photoreceptors [31, 38, 39, 40, 41] and RPE cells [42, 43]. Various PEDF‐mediated mechanisms that promote cell survival have been identified, including the activation of STAT3 [44], interference with calcium pumps leading to decreased intracellular calcium levels [45], anti‐apoptotic effects through modulation of Bcl2 and AIF [40], as well as its influence on glucose metabolism [46, 47], lipid metabolism [48], and oxidative‐stress pathways [49, 50].

Another mechanism by which PEDF promoted photoreceptor survival in our study is through its stabilizing effects on CNV vessels, which in turn improved the blood supply to the overlying tissues. Similar to the results demonstrated in an ex vivo rat eye model [31], in the present study PEDF increased the cross‐section area of the vessel lumen, maintaining the vessels open. Interestingly, in our model bevacizumab did not exhibit a stabilizing effect on the vessels but did not show an induction of platelet activation, degranulation of thrombocytes and neutrophils, or immune complex formation as demonstrated in monkeys [51].

In patients, CNV formation is closely associated with excessive ECM deposition and remodeling, which can eventually progress into a fibrovascular complex and macular fibrosis [52, 53]. In this study, we also observed excessive accumulation of ECM in the CNV area. However, a closer ultrastructural examination of the ECM layers around the CNV vessels revealed that in non‐PEDF‐treated animals, the ECM was thickened and displayed a disorganized fiber structure and clustered appearance (Figures 4 and 6a). In contrast, in the PEDF‐ and PEDF‐bevacizumab‐treated groups, the thickness of the ECM around the CNV vessels was not significantly different from that of choriocapillaris vessels (Figure 6b). PEDF also refined ECM structure, reducing areas with disordered fiber organization and ECM clusters (Figures 4 and 6a), which is consistent with previous reports of PEDF's ECM modulating abilities [54, 55, 56, 57], vessel structure refinement, and promotion of tissue regeneration [29].

Pericytes are crucial for the organized growth, maintenance, stabilization, functionality, and permeability of blood vessels [58, 59]. Their loss is a characteristic feature of pathological CNV formation in both wet AMD [60, 61, 62] and diabetic retinopathy [63].

Interestingly, pericytes can mediate partial resistance to anti‐VEGF therapies, reducing their therapeutic effectiveness [64, 65]. This has spurred the development of anti‐pericyte therapies, such as the combination of Pegpleranib (Fovista), an anti‐PDGF therapeutic, with ranibizumab, an anti‐VEGF agent. Despite its advancement to a phase 2b clinical trial, this combination did not demonstrate superior visual acuity compared to anti‐VEGF monotherapy [66]. Other anti‐pericyte approaches for CNV are still under investigation [67, 68, 69], which is understandable given the goal of completely blocking and removing CNV vessels. In contrast, our innovative strategy focuses on stabilizing healthy CNV vessels, making pericyte coverage essential. Multiple studies have indicated that PEDF enhances pericyte survival and promotes their association with endothelial cells [49, 70, 71]. Our study's results (Figure 7b) support that PEDF can effectively contribute to a pericyte‐driven stabilization.

Another interesting CNV treatment approach by pericyte stabilization is faricimab, a bispecific monoclonal antibody that simultaneously targets Ang‐2, which induces pericyte apoptosis and detachment, and VEGF. This treatment was recently tested in the JR5558 mutant mouse model, which spontaneously develops CNV [72]. Anti‐Ang‐2 treatment resulted in improved endothelial cell–pericyte interactions and reduced vascular leakage [73]. Phase 3 clinical trials of faricimab have shown it to be non‐inferior compared to anti‐VEGF treatment in terms of efficacy, durability, and safety for treatment‐naïve AMD and diabetic macular edema, and it has demonstrated superior efficacy in treatment‐resistant cases [74].

Fenestrations are critical structural features of vessels that significantly contribute to their functionality. The choriocapillaris vessels are characterized by a high degree of fenestration [75, 76]. Several studies have demonstrated a positive correlation between VEGF expression and the presence of fenestrations [77, 78]. Conversely, treatment with anti‐VEGF therapeutics reduced fenestration in the choriocapillaris [79] and ciliary blood vessels [80] of monkeys. This structural characteristic is particularly relevant in the context of CNV. Alterations in fenestrations in pathological conditions and their response to various treatments may have significant implications for vessel functionality and the health of surrounding tissues.

In our study, we observed that in untreated, vehicle‐ and bevacizumab‐treated eyes, most CNV vessels lacked fenestrations (Figure 8b). This finding is consistent with previous studies on CNV vessels from choroidal neovascular membranes of patients who underwent submacular surgery, which reported unexpectedly low fenestration levels [1]. The same study showed that fenestration of CNV vessels in patients treated with bevacizumab before surgery was not distinguishable from that of untreated patients. Similar results were reported for the quantification of fenestration of vessels from AMD patients in Grebe et al. [81]. A higher number of vessels with fenestrations and therefore enhanced functionality (gas and metabolite exchange) led to better supply and potentially contributed to the improved survival of overlying tissues observed in these treatment groups. The underlying mechanisms of PEDF's effects on fenestration of vessels are not well understood, but a positive correlation between PEDF and fenestration factors HARE‐Y20 (stabilin‐2) and PV‐1 has been reported [71].

Taken together, in our model, PEDF monotherapy as well as PEDF combined with bevacizumab showed similar effects on photoreceptor preservation and CNV vessel structure stabilization. Unfortunately, functional outcomes were not assessed here; a follow‐up study including ERG is planned.

PEDF is a promising candidate for CNV treatment by protecting photoreceptors via CNV stabilization rather than CNV reduction as with anti‐VEGF drugs. Furthermore, it is safe at doses up to 100 μg per eye [32]. But the effects of long‐term treatments or possible development of resistance have not yet been analyzed here.

In patients, the anti‐VEGF treatment is the established standard therapy, and it is known to reduced leakage. Although, it was not possible to investigate the effect of PEDF on leaky CNV vessels in our study, we demonstrated that PEDF did not show any adverse effects in combination with bevacizumab and these two are therefore combinable. Therefore, it could be a valuable adjunct to current anti‐VEGF treatments particularly in patients with leaky CNV and offer an alternative therapeutic option for anti‐VEGF non responders.

## 5 Limitations of the Study

This study has several limitations that should be considered when interpreting the findings and that point toward important directions for future research.

First, the evaluation of photoreceptor protection was based exclusively on structural measurements of ONL area. While these data suggest preservation, no direct assessment of apoptosis was performed. The absence of TUNEL staining or alternative assays of cell death means that “photoreceptor survival” should be interpreted as an indirect inference rather than a definitive demonstration. Incorporating such methods in future studies would provide stronger mechanistic evidence.

Second, this work does not demonstrate functional improvement of either the CNV or the retina. The evidence for PEDF‐mediated CNV stabilization derives primarily from electron microscopy, which is a powerful technique for visualizing ultrastructural changes of the choriocapillaris but does not capture functional outcomes. Moreover, the relatively small number of samples analyzed by EM, although standard for this labor‐intensive method, further underscores the need for complementary approaches with higher throughput.

Together, these limitations highlight the importance of integrating structural, molecular, and functional readouts in future studies. Such approaches will be critical to fully establish the therapeutic potential of PEDF in CNV.

## Author Contributions

A.V.T. and S.J. wrote the manuscript; A.V.T., U.S., and S.J. conducted the experiments. A.V.T., L.X., and S.J. acquired and analyzed the data. U.S. and S.J. designed the study. All authors were involved in drafting and revising the manuscript.

## Conflicts of Interest

The authors declare no conflicts of interest.

## Supporting information


**Figure S1:** The upper panel shows a representative fluorescein (FA) and indocyanine green (ICG) angiography image from a rat eye three weeks after AAV.VEGF vector injection. The hyperfluorescent (bright) ring‐shaped CNV‐area is clearly visible in the central part of each of the images. The lower panel shows close up images of the CNV‐area, which appears in both angiographies as an ill‐defined hyperfluorescent area without leakage or pooling of the dyes.

**Figure S2:** The upper part shows a multi‐image alignment (MIA) consisting of 89 images (3000× magnification) showing the CNV‐area of an untreated rat eye seven weeks post‐VEGF vector injection. In the red rectangle a CNV vessel can be identified. The lower part shows an electron micrograph of the CNV vessel in the red rectangle (7000 x magnification). A large ECM‐cluster is circled in blue, blue c ‐collagen layer, blue d‐ layer of electron dense material, n‐ nucleus, e‐ erythrocytes, yellow m‐ melanin granules.

## Data Availability

The data that support the findings of this study are available in the methods of this article.
