# Supplementary figures and images for: Pigment Epithelium‐Derived Factor (PEDF)‐Based Therapy Induced Photoreceptor Survival by Stabilizing Choroidal Neovessels in a VEGF Overexpression CNV Rat Model
Source: FASEB J. 2025 Oct 9;39(19):e71113. doi: 10.1096/fj.202501704RR (PMC12510281; doi:10.1096/fj.202501704RR)

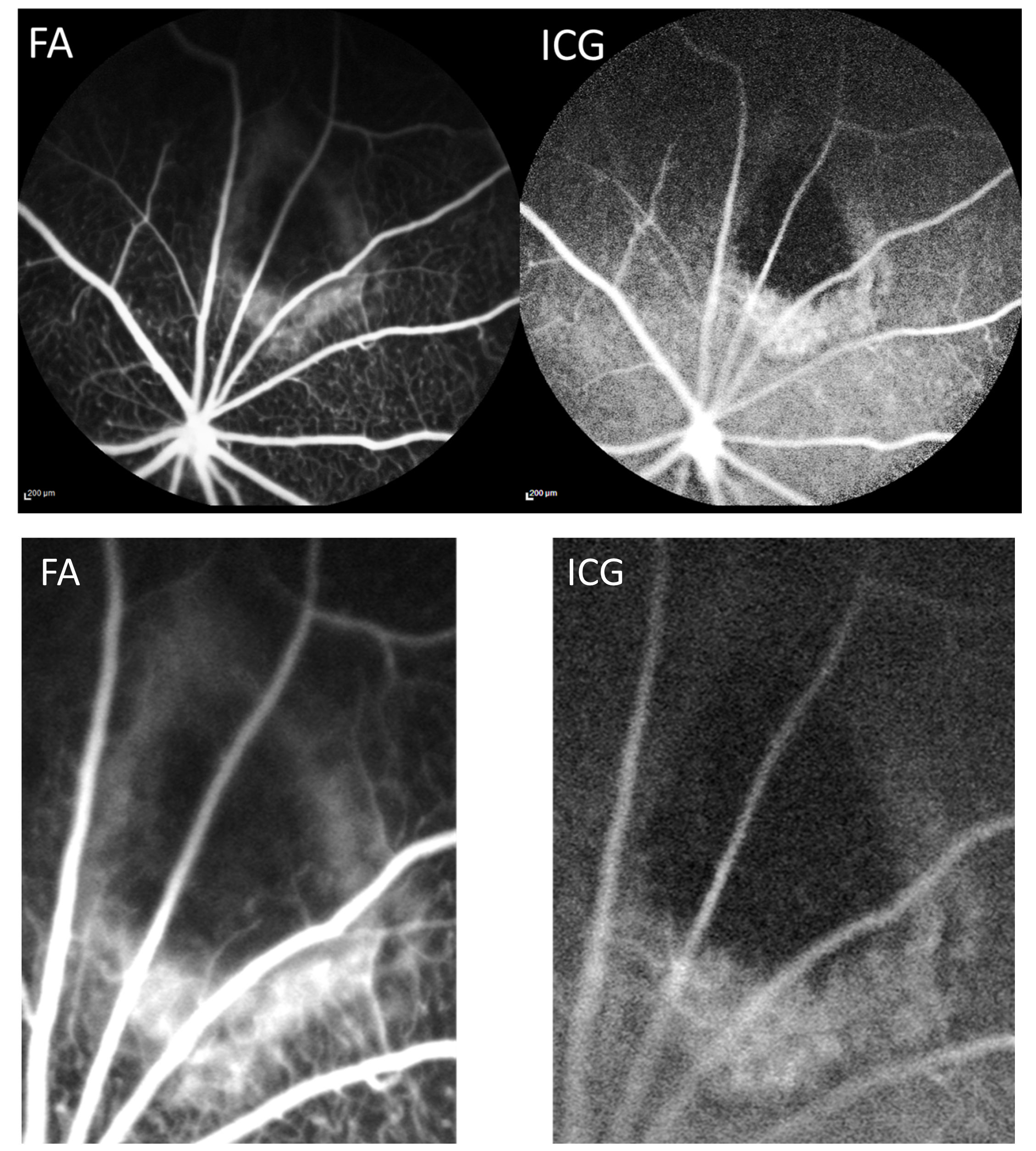

Supplement: Supplementary file 1 — Figure S1: The upper panel shows a representative fluorescein (FA) and indocyanine green (ICG) angiography image from a rat eye three weeks after AAV.VEGF vector injection. The hyperfluorescent (bright) ring‐shaped CNV‐area is clearly visible in the central part of each of the images. The lower panel shows close up images of the CNV‐area, which appears in both angiographies as an ill‐defined hyperfluorescent area without leakage or pooling of the dyes. [file FSB2-39-e71113-s001.tif]

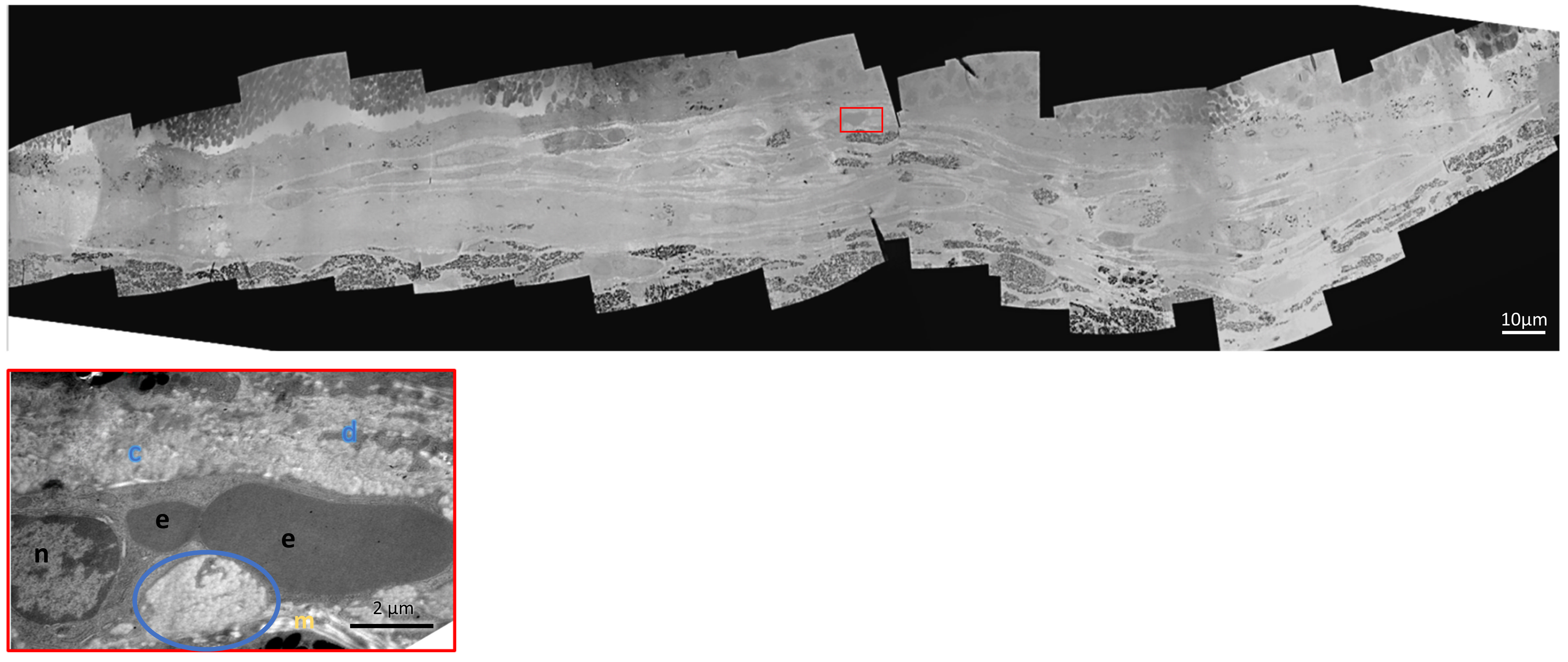

Supplement: Supplementary file 2 — Figure S2: The upper part shows a multi‐image alignment (MIA) consisting of 89 images (3000× magnification) showing the CNV‐area of an untreated rat eye seven weeks post‐VEGF vector injection. In the red rectangle a CNV vessel can be identified. The lower part shows an electron micrograph of the CNV vessel in the red rectangle (7000 x magnification). A large ECM‐cluster is circled in blue, blue c ‐collagen layer, blue d‐ layer of electron dense material, n‐ nucleus, e‐ erythrocytes, yellow m‐ melanin granules. [file FSB2-39-e71113-s002.tif]
